# Views of primary care physicians and rheumatologists regarding screening and treatment of hyperlipidemia among patients with rheumatoid arthritis

**DOI:** 10.1186/s41927-020-0112-5

**Published:** 2020-03-05

**Authors:** Iris Navarro-Millán, Anna Cornelius-Schecter, Ronan J. O’Beirne, Melanie S. Morris, Geyanne E. Lui, Susan M. Goodman, Andrea L. Cherrington, Liana Fraenkel, Jeffrey R. Curtis, Monika M. Safford

**Affiliations:** 1 Division of General Internal Medicine, Weill Cornell Medicine, 420 E 70th Street, LH-363, New York, NY 10021 USA; 2 Division of Rheumatology, Hospital for Special Surgery, 535 E 70th Street, New York, NY 10021 USA; 3 University of Alabama at Birmingham, Birmingham, AL USA; 4 Yale University, New Haven, CT USA; 5 Berkshire Health Systems, Pittsfield, MA USA

**Keywords:** Rheumatoid arthritis, Physician perspective, Hyperlipidemia, Cardiovascular disease, Statins

## Abstract

**Background:**

Despite high risk for cardiovascular disease (CVD) mortality, screening and treatment of hyperlipidemia in patients with rheumatoid arthritis (RA) is suboptimal. We asked primary care physicians (PCPs) and rheumatologists to identify barriers to screening and treatment for hyperlipidemia among patients with RA.

**Methods:**

We recruited rheumatologists and PCPs nationally to participate in separate moderated structured group teleconference discussions using the nominal group technique. Participants in each group generated lists of barriers to screening and treatment for hyperlipidemia in patients with RA, then each selected the three most important barriers from this list. The resulting barriers were organized into physician-, patient- and system-level barriers, informed by the socioecological framework.

**Results:**

Twenty-seven rheumatologists participated in a total of 3 groups (group size ranged from 7 to 11) and twenty PCPs participated in a total of 3 groups (group size ranged from 4 to 9). Rheumatologists prioritized physician level barriers (e.g. ‘ownership’ of hyperlipidemia screening and treatment), whereas PCPs prioritized patient-level barriers (e.g. complexity of RA and its treatments).

**Conclusion:**

Rheumatologists were conflicted about whether treatment of CVD risk among patients with RA should fall within the role of the rheumatologist or the PCP. All participating PCPs agreed that CVD risk reduction was within their role. Factors that influenced PCPs’ decisions for screening and treatment for CVD risk in patients with RA were mainly related to their concern about how treatment for CVD risk could influence RA symptomatology (myalgia from statins) or how inflammation from RA and RA medications influences lipid profiles.

## Background

Patients with rheumatoid arthritis (RA) are at high risk for cardiovascular disease (CVD) [1–5], and experience approximately 50% higher CVD mortality compared to the general population [5]. While this increased CVD risk may in part be due to accelerated atherosclerosis from inflammation related to RA [6], clinical practice guidelines recommend that the cornerstone of CVD risk reduction in this population should follow a similar approach as that for the general population [7, 8]. This approach relies on assessment of 10-year CVD risk, which requires assessment of lipid levels, along with blood pressure, diabetes status, and smoking status.

Unfortunately, ours and others’ previous work have shown that lipid assessment among patients with RA is as low as 37% [9–11]. Although the likelihood of cholesterol testing increases if patients are followed by both primary care providers (PCP) and rheumatologists [9, 10], some RA patients do not see a PCP, decreasing the likelihood of cholesterol testing [9–11]. Even among patients with RA and known coronary heart disease, only 64% of patients had a cholesterol test [11].

The reasons for this lack of lipid assessment are not well understood. One qualitative study among rheumatologists, primary care providers (PCPs), and patients with RA found that lack of knowledge about increased CVD risk in the population was common [12]. Since their focus was on CVD risk reduction overall (e.g. diabetes screening, hypertension, smoking, cholesterol level screening), thus it could not shed light on the low rates of lipid testing specifically [12]. The goal of this study was to learn about barriers to lipid testing specifically among RA patients, from the perspective of both rheumatologists and PCPs.

## Methods

### Study participants

We invited PCPs (general internists or family medicine physicians) and rheumatologists by email to participate in one of six online nominal groups held in October and December 2017. Physicians were eligible if they treated patients with RA (self-reported) and available to participate in a nominal group for around 90 min. We sent email invitations to rheumatologists who were members of the American College of Rheumatology (ACR). These members of the ACR are part of a manually curated list maintained by one of the authors. We also invited rheumatology faculty at two medical centers (University of Alabama at Birmingham (UAB) in Birmingham, AL and the Hospital for Special Surgery in New York, NY to participate. We recruited PCPs from faculty at Weill Cornell Medicine (WCM), Columbia University, and members of the Deep South Continuing Medical Education (CME) Network. The Deep South Network includes approximately 1200 physicians, physician assistants, and nurses who maintain ongoing relationships with the UAB Division of CME. Network members were mainly from Alabama and Mississippi with a minority residing in an additional 32 states and 11 countries.

### Nominal group sessions

We used the socioecological framework to design and guide the analysis of the study [13]. This model posits that interpersonal, organizational, community, and public policy factors may be targeted to effect changes in the structure and process of healthcare to influence health outcomes.

The nominal group technique is a semi-quantitative method that uses a structured group process designed to elicit a prioritized list of responses to a question [14, 15]. Sample size calculations are not used in this method, but recruitment and data collection continues until no new ideas emerge, indicating thematic saturation. We conducted six online nominal group sessions, three for rheumatologists and three for PCPs. A trained moderator led each session, assisted by a scribe. Participants called into a conference call line and logged into a website designed to support nominal group sessions. After describing the purpose of the study and the nominal group procedure, the moderator read the question for the group’s consideration, which was also displayed on the website. Each group considered two questions, which were addressed separately: *“What are some of the things that make it difficult for you to screen for hyperlipidemia in your RA patients?”* and *“What are some of the reasons why you may choose not to treat these patients for hyperlipidemia?”*

Each of the six groups then generated a list of statements in response to each question that were transcribed verbatim. Each participant first wrote down his or her responses during a 5-min silent period. They were then asked to contribute a single idea expressed as a phrase or brief sentence in a round-robin format. The contributions were captured verbatim by the scribe and displayed as a list on the participants’ screens. This process continued until the group felt that all significant ideas had been captured. All listed items were then reviewed to ensure that all participants had a shared understanding of the items. Following review, participants were asked to select the three most important items on the list in order of importance from their own perspective. We retained this rank order for further analysis. After the ranked list was generated, the process was repeated for the second question. On completion of the nominal groups, participants completed a brief demographic questionnaire. Each nominal group session lasted approximately 90 min.

### Analysis

The resulting items from each group were aggregated by the investigators (INM, ACS, GL, and MMS) into common categories informed by the constructs of the socioecological model. In order to summarize the findings, we created a relative importance score for each item generated during the nominal group session. For each participant, we assigned a weight of 3 points for the item selected as the most important, 2 for the second most important, 1 for the third most important, and 0 for all other items. The points were then summed for each item over the participants in each group and divided by the number of participants in each group. This resulted in each item receiving a weighted sum of priority points that could be combined across groups while accounting for the different number of individuals in each group (percentage of total votes). The institutional review board from WCM and UAB approved the study protocol.

## Results

During recruitment, two primary care physicians declined to participate because of the low number of RA patients they followed. All rheumatologists that participated in the study reported seeing patients with RA regularly. The final sample was 27 rheumatologists who participated in three groups of eleven, eight, and seven participants each, respectively; and 20 PCPs who participated in three groups of seven, four, and nine participants each, respectively. After analyzing three groups for each type of participant, no new ideas emerged, indicating thematic saturation and obviating the need for additional nominal groups. Table 1 shows demographic information about the participants. Fifty-six percent of rheumatologists and 20% of PCPs were over age 50. Sixty percent of rheumatologists and 40% of PCPs were women, and both groups of physicians were mostly White (64% of rheumatologists and 60% of PCPs). Approximately half of both rheumatologists and PCPs resided in the Northeast of the United States.

**Table 1:** Demographic of primary care physicians (PCPs) and rheumatologists that participated in the nominal groups

Characteristic	Rheumatologist^a^ *N* = 25	PCP^b^ *N* = 20
Age, N (%)		
> 50 years	14 (56)	5 (25)
Sex, N (%)		
Male	10 (40)	12 (60)
Race/Ethnicity, N (%)		
White	16 (64)	12 (60)
Non-White	9 (36)	8 (40)
Geographic Region, N (%)		
Northeast	13 (48)	10 (50)
Midwest	3 (11)	2 (10)
South	8 (30)	8 (40)
West	3 (11)	0 (0)
Is screening for hyperlipidemia your responsibility? N^a^ (%)		
Yes	9 (36)	20 (100)
No	12 (48)	0 (0)
Uncertain	4 (16)	0 (0)

^a^Two rheumatologists did not complete the demographic survey

^b^Four PCP were family medicine, 16 general internal medicine

The three main categories of barriers to screening and treatment based on the socioecological framework consisted of patient-, physician-, and system-level barriers (Tables 2 and 3). The ideas generated during the nominal groups are shown in Additional file 1: Table S1, Additional file 2: Table S2, Additional file 3: Table S3 and Additional file 4: Table S4 (verbatim).

**Table 2:** Physicians’ barriers to screen patients with rheumatoid arthritis for hyperlipidemia with their respective priority votes

Level	Sub-level	Rheumatologist Votes (%)^a^	PCP Votes (%)^a^
Physician Level	Total Votes, %	82.7	42.5
	Lack of time	34.0	1.7
	Conflict regarding ownership of hyperlipidemia screening	25.9	10.8
	Lack of training and knowledge of hyperlipidemia guidelines	17.9	19.2
	Focus only on RA	4.9	–
	Physician prioritization of RA symptomology over preventive measures	–	10.0
	Lack of physician knowledge about RA	–	0.8
Patient Level	Total Votes, %	7.4	44.2
	Complexity of RA and its treatment	2.5	9.2
	Patient prioritization of RA symptomology over preventive measures	2.5	9.2
	Patient expectations	1.8	–
	Patient already on multiple medications	0.6	0.0
	Side effects of RA medications and RA drug interactions	0.0	–
	Comorbidities	0.0	–
	Patient’s barriers with transportation	0.0	–
	Multiple blood draws	0.0	8.3
	Side effects of statins and drug interactions with statins	0.0	5.0
	Poor patient compliance with medical care	0.0	9.2
	Patients’ lack of awareness of CVD risk	–	3.3
System Level	Total Votes, %	9.9	13.3
	Lack of care coordination	6.8	11.7
	Financial barriers (limited insurance coverage, cost of repeating labs)	3.1	0.0
	Lack of financial incentive for screening	–	1.7

*RA* Rheumatoid arthritis, *CVD* Cardiovascular disease. 0% = that sub-level emerged during the brainstorming session but did not receive votes. “--” = the sub-level did not emerge in the respective group

^a^Total votes are calculated based on the number of participating physicians. Each physician had a total of 6 votes (3 for the most important, 2 votes for the second most important, and 1 for the third most important statement). Hence, 27 rheumatologist participated × 6 votes each = 162 votes; 20 PCPs participated × 6 votes = 120 votes

**Table 3:** Physicians’ barriers to treat hyperlipidemia among patients with rheumatoid arthritis with their respective priority votes

Level	Sub-level	Rheumatologist Votes (%)^a^	PCP Votes (%)^a^
Physician Level	Total Votes, %	87.0	25.0
	Conflict regarding ownership of hyperlipidemia management	37.7	0.0
	Lack of training and knowledge of hyperlipidemia guidelines	32.1	18.3
	Lack of time	11.1	0.8
	Focus only on RA	6.2	–
	Prioritize non-pharmacologic measures (diet and exercise)	–	4.2
	Difficulty implementing lifestyle modifications for patients with pain	–	1.7
Patient Level	Total Votes, %	5.6	69.2
	Side effects of statins	2.5	42.5
	Patient already on multiple medications	2.5	8.3
	Side effects of RA medications and RA drug interactions	0.6	–
	Comorbidities	–	6.7
	Complexity of RA and its treatment	–	5.8
	Patients’ lack of awareness of CVD risk	–	1.7
	Priority of RA symptomology over preventive measures	–	4.2
System Level	Total Votes, %	7.4%	5.8
	Financial barriers (limited insurance coverage, cost of additional medications, cost of repeating labs)	6.2%	0.0
	Limited clinic staff support	1.2%	–
	Lack of care coordination	–	5.8

*RA* Rheumatoid arthritis, *CVD* Cardiovascular disease. 0% = that sub-level emerged during the brainstorming session but did not receive votes. “--” = the sub-level did not emerge in the respective group

^a^Total votes are calculated based on the number of participating physicians. Each physician had a total of 6 votes (3 for the most important, 2 votes for the second most important, and 1 for the third most important statement). Hence, 27 rheumatologist participated × 6 votes each = 162 votes; 20 PCPs participated × 6 votes = 120 votes

### Rheumatologists

For screening, physician-level barriers received 82.7% of total votes (Table 2). The highest ranked sub-level in the physician level was “lack of time” with 34.0% of total votes. The items under the “lack of time” sub-level were related to the difficulties of managing other conditions and addressing patients’ questions about CVD risk in addition to those regarding RA (e.g. medication side effects, disease activity) (Additional file 1: Table S1). Rheumatologists also perceived conflict regarding ownership of hyperlipidemia screening (25.9% of total votes) and that they lack training and knowledge of hyperlipidemia guidelines (17.9% of total votes). They also expressed that, as rheumatologists, their main focus is to treat RA, and by doing so they are also controlling CVD risk (“focus only in RA” sub-level, which received 4.9% of total votes). Only 7.4% of total votes referred to patient-level barriers, and 9.9% of total votes to system-level barriers, with 6.8% of votes assigned to “lack of care coordination”.

For treatment of hyperlipidemia, the physician-level barriers received 87.0% of the total votes, including 37.7% for “conflict regarding ownership of hyperlipidemia management,” 32.1% for “lack of training and knowledge of hyperlipidemia guidelines” (Table 3). The patient-level barriers received only 5.6% and system-level only 7.4% of the total votes.

### Primary care physicians

Among PCPs, for screening, the physician-level barriers received 42.5% of total votes, with sub-levels led by “Lack of training and knowledge of hyperlipidemia guidelines” (19.2% of total votes) (Table 2). The patient-level barriers received 44.2% of votes, with sub-levels of “RA complexity”, “patient prioritization of RA symptomatology over preventive measures”, and “poor patient compliance” each receiving 9.2% of total votes. The system-level barriers received 13.3% of the total votes, with “lack of care coordination” receiving 11.7% of total votes.

For treatment, the physician-level barriers received 25% of the total votes, with “Lack of training and knowledge of hyperlipidemia guidelines” receiving 18.3% (Table 3). The patient-level barriers received 69.9% of the total votes, with “side effects of statins” receiving 42.5% of the total votes. The system-level barriers received 5.8% of total votes, all in the “lack of care coordination” sub-level.

## Discussion

We elicited barriers for screening and treatment of hyperlipidemia among patients with RA from a national sample of rheumatologists and PCPs. The findings from this study allowed us to understand possible reasons for the current low screening and treatment rates for hyperlipidemia among patients with RA. Just over one third of the rheumatologists thought that CVD risk prevention was their responsibility. For both screening and treatment, rheumatologists perceived barriers mainly at the physician level that included aspects such as conflict regarding ownership of hyperlipidemia, lack of familiarity with cholesterol management guidelines, and lack of time. The perspectives of PCPs and rheumatologists at the physician level for screening for hyperlipidemia were similar and included conflict regarding the different providers’ role and responsibility regarding screening and treating for cardiovascular disease, and lack of knowledge of the cholesterol treatment guidelines. For treatment, most of the barriers for PCPs were at the patient level, including patients’ concern about statin side effects, multiple medications, and that patients may prioritize treatment of RA over preventive CVD measures. Both rheumatologists and PCPs cited challenges with care coordination as a barrier for screening and treatment of hyperlipidemia among patients with RA. These findings suggest possible reasons behind prior observations that many patients with RA do not receive cholesterol testing as part of an overall CVD risk reduction strategy.

Several quantitative and qualitative studies over the past 30 years have shown that primary CVD prevention by both specialists and general practitioners has been challenging [10, 12, 16, 17]. The common barriers in these studies have been lack of understanding of CVD prevention guidelines, uncertainty about initiation of primary preventive measures for either asymptomatic or low risk patients, who should assume the role of CVD prevention (the specialist or generalist), and how best to coordinate care between providers [12, 17]. As to the barrier of education, our study showed that there are existing challenges in interpreting and disseminating these guidelines and this will continue to be a challenge since some physicians, mainly specialists, do not see this type of clinical management within their purview and do not keep up with guideline updates [12, 17].

Our findings suggest that engaging PCPs may yield the higher probability of achieving lipid testing as PCPs thought of this process as lying within their scope of practice. This is consistent with previous studies that demonstrated that screening was 55% higher in patients with RA who visited a PCP vs. visiting a rheumatologist only [9]. Patients with RA may want to engage in discussions with their rheumatologist about lipid testing due to the frequency of visits with the rheumatologist as part of RA medication surveillance. However, we found that few rheumatologists may agree to manage CVD risk by prescribing statins. This finding underscores the need for primary care among patients with RA. A systematic review of health information technology (HIT) interventions showed that the most efficacious interventions to increase CVD risk screening involved not only providers but also patients [18]. These consisted of a combination of clinical decision support and patient education among community primary care clinics for general primary prevention [18–20]. Our results and that of others support the need for patients with RA to have a PCP, which might not only increase the likelihood of CVD screening itself but also enable the implementation of interventions that can increase CVD risk screening and reduction.

The strengths of this study include the use of the nominal group technique to elicit and prioritize barriers that PCPs and rheumatologists perceive to screening and treating hyperlipidemia in patients with RA. This technique is suited for understanding multiple perspectives on an issue, eliciting responses from not just those with strong opinions or personalities (a limitation of other forms of qualitative research such as focus groups), and prioritizing root causes of a problem [14, 21]. This method has demonstrated validity, and considers all participants’ views equally [22]. We recruited both community and academic rheumatologists and PCP nationally, which helps to include diverse views that are not limited to a single center or region. The study’s limitations include the relatively modest sample size with potentially limited generalizability, which may have been somewhat mitigated by our national sampling strategy. This study was conducted in the United States, and healthcare systems may differ in other countries, possibly limiting generalizability.

## Conclusions

Many rheumatologists in this study expressed that management of CVD risk among their patients with RA does not fall within their role. In contrast, PCPs agreed that CVD risk management was within their purview, but they expressed concern about lack of knowledge about how treatment for CVD risk could influence RA symptomatology (myalgia from statins) or how inflammation from RA and RA medications influence lipid profiles. These findings improve our understanding of the reasons for low lipid testing and CVD risk screening in patients with RA. There is a need for interventions that improve care coordination among physicians, educate patients with RA about the need to directly address CVD risk screening with their rheumatologist, and be prepared to see a PCP regularly for follow up treatment and management of CVD risk.

## Supplementary information

**Additional file 1: Table S1.** Rheumatologists’ responses to “What are some of the things that make it difficult for you to screen for hyperlipidemia in your RA patients?”

**Additional file 2: Table S2.** Primary care physicians’ responses to “What are some of the things that make it difficult for you to screen for hyperlipidemia in your RA patients?”

**Additional file 3: Table S3.** Rheumatologists’ responses to “What are some of the reasons why you may choose not to treat these patients for hyperlipidemia?”

**Additional file 4: Table S4.** Primary care physicians’ responses to “What are some of the reasons why you may choose not to treat these patients for hyperlipidemia?”

## Data Availability

All data generated or analyzed during this study are included in this published article.

## Abbreviations

ACR: American College of Rheumatology
CME: Continuing medical education
CRP: C-reactive protein
CVD: Cardiovascular disease
DM: Diabetes mellitus
ESR: Erythrocyte sedimentation rate
MI: Myocardial infarction
PCPs: Primary care providers
RA: Rheumatoid arthritis
UAB: University of Alabama at Birmingham
WCM: Weill Cornell Medicine
