# Supplementary material for: Views of primary care physicians and rheumatologists regarding screening and treatment of hyperlipidemia among patients with rheumatoid arthritis
Source: BMC Rheumatol. 2020 Mar 5;4:14. doi: 10.1186/s41927-020-0112-5 (PMC7057468; doi:10.1186/s41927-020-0112-5)
Supplement: Supplementary file 1 — Additional file 1: Table S1. Rheumatologists’ responses to “What are some of the things that make it difficult for you to screen for hyperlipidemia in your RA patients?” [file 41927_2020_112_MOESM1_ESM.docx]

**Supplemental Table 1.** Rheumatologists’ responses to “What are some of the things that make it difficult for you to screen for hyperlipidemia in your RA patients?”

| **Group** | **Statement** | **Category** | **Sub-category** | **% of total points** |
| --- | --- | --- | --- | --- |
| 3 | For older patients (75 or more) the risk to benefit ratio for statin use is high | Patient Level | Comorbidities | 0.0% |
| 3 | Overall medical complexity would lead you to avoid taking the step of screening | Patient Level | Complexity of RA and its treatment | 1.9% |
| 3 | Don't want to add another complicating factor of the risk for myopathy from statins | Patient Level | Complexity of RA and its treatment | 0.6% |
| 3 | Patient compliance | Patient Level | Poor patient compliance with medical care | 0.0% |
| 1 | Failure of patient to follow up with PCP or Cardiologist when instructed to do so | Patient Level | Poor patient compliance with medical care | 0.0% |
| 2 | Distance a patient might have to travel to have screening done | Patient Level | Patient's barriers with transportation | 0.0% |
| 2 | Patient's willingness to get labs | Patient Level | Multiple blood draws | 0.0% |
| 2 | Patients expect that their PCP or cardiologist would deal with CVD risk | Patient Level | Patient expectations | 1.2% |
| 1 | Patients assume PCPs will address cholesterol [and don't understand why tests are/aren't ordered] | Patient Level | Patient expectations | 0.6% |
| 1 | Patient preference to focus on joint pain instead of discussing CV risk | Patient Level | Patient prioritization of RA symptomology over preventive measures | 2.5% |
| 3 | Limited understanding on the part of the patient - don't associate CV risk with RA | Patient Level | Patient prioritization of RA symptomology over preventive measures | 0.0% |
| 3 | Patient may be reluctant to add an additional medication | Patient Level | Patient already on multiple medications | 0.6% |
| 3 | Patient fear of muscle side effects | Patient Level | Side effects of statins and drug interactions with statins | 0.0% |
| 3 | Methotrexate and some statins can cause liver abnormalities | Patient Level | Side effects of RA medications and RA drug interactions | 0.0% |
| 2 | Frequently patients are already on hyperlipidemia medicine and don't feel like it is up to me to determine if it is well managed | Physician Level | Conflict regarding ownership of hyperlipidemia management | 0.6% |
| 1 | Uncertain that it is appropriate to address lipid levels as a rheumatologist [leave to primary care or cardiology] | Physician Level | Conflict regarding ownership of hyperlipidemia management | 3.1% |
| 1 | Don't want to "enter the door" to managing general cardiac care, stress testing, etc. | Physician Level | Conflict regarding ownership of hyperlipidemia management | 3.1% |
| 1 | Expectation that other physicians are better prepared to manage comorbidities | Physician Level | Conflict regarding ownership of hyperlipidemia management | 1.9% |
| 1 | PCs get angry in some instances and perceive a "territorial offense" which can impact future referrals | Physician Level | Conflict regarding ownership of hyperlipidemia management | 1.2% |
| 1 | Lack of awareness that rheums should monitor lipid levels | Physician Level | Conflict regarding ownership of hyperlipidemia management | 0.0% |
| 1 | Lack of knowledge among PCPs about elevated risk for hyperlipidemia | Physician Level | Conflict regarding ownership of hyperlipidemia management | 0.0% |
| 2 | Rheumatologists want PCP's to take ownership of lipid management | Physician Level | Conflict regarding ownership of hyperlipidemia management | 4.9% |
| 2 | Expectation that it was already checked and addressed during the primary care visit | Physician Level | Conflict regarding ownership of hyperlipidemia management | 2.5% |
| 2 | Don't want to step on the toes of the referring physician | Physician Level | Conflict regarding ownership of hyperlipidemia management | 1.9% |
| 3 | CVD risk and screening for it is usually the responsibility of the PCP or other specialists | Physician Level | Conflict regarding ownership of hyperlipidemia management | 6.2% |
| 3 | As a rheumatologist, don't want to step on the toes of the PCP (definition of who the primary caregiver is) | Physician Level | Conflict regarding ownership of hyperlipidemia management | 0.6% |
| 2 | We have too much responsibility already | Physician Level | Lack of time | 0.6% |
| 2 | Many other issue take precedence and might not think about asking about cardio history | Physician Level | Lack of time | 1.9% |
| 3 | Competing demands on time to discuss another problem related to their RA | Physician Level | Lack of time | 5.6% |
| 1 | Hard to manage complex chronic disease to start with, and other tasks compete | Physician Level | Lack of time | 6.2% |
| 1 | Lack of time to address all aspect of the disease; hard to stratify for cardiovascular risk | Physician Level | Lack of time | 8.6% |
| 1 | Not enough time answer all questions on cardiac risk | Physician Level | Lack of time | 1.9% |
| 2 | Time constraints don't allow me to examine all risk factors | Physician Level | Lack of time | 4.3% |
| 3 | Time to order a test and discuss results is not available in a regular patient visit | Physician Level | Lack of time | 4.9% |
| 3 | For younger patients, may overlook the need for the test (in the absence of other risk factors) | Physician Level | Lack of training and knowledge of hyperlipidemia guidelines | 0.0% |
| 1 | Patients often arrive without having fasted | Physician Level | Lack of training and knowledge of hyperlipidemia guidelines | 1.9% |
| 2 | It is often inconvenient for the patient (may not have fasted) | Physician Level | Lack of training and knowledge of hyperlipidemia guidelines | 0.6% |
| 1 | Many Rheums lack current training on how to monitor and manage hyperlipidemia | Physician Level | Lack of training and knowledge of hyperlipidemia guidelines | 4.3% |
| 1 | Guidelines for evaluating risk are not adequate | Physician Level | Lack of training and knowledge of hyperlipidemia guidelines | 2.5% |
| 2 | Lack of education on current lipid management | Physician Level | Lack of training and knowledge of hyperlipidemia guidelines | 6.8% |
| 3 | Don't feel comfortable with their knowledge of current guidelines concerning hyperlipidemia | Physician Level | Lack of training and knowledge of hyperlipidemia guidelines | 1.9% |
| 3 | Perceived lack of guidelines for recommending lipid screening in RA patients | Physician Level | Lack of training and knowledge of hyperlipidemia guidelines | 0.0% |
| 1 | Rheumatologists focus on RA symptoms [tunnel vision] | Physician Level | Focus only in RA | 3.1% |
| 2 | As a rheumatologist, must address the condition of RA | Physician Level | Focus only in RA | 1.9% |
| 3 | If not starting anti TNF's, then usually don't screen | Physician Level | Focus only in RA | 0.0% |
| 1 | Patients often say PC checked cholesterol, but it is not always accessible from PC and difficult to re-order [insurance rules and patient reluctance] | System Level | Lack of care coordination | 0.0% |
| 2 | Communication between physicians can be inadequate | System Level | Lack of care coordination | 1.9% |
| 2 | Ability to share labs with patients PCP | System Level | Lack of care coordination | 1.9% |
| 3 | Primary care may have already checked it (but the rheumatologist may not know and it is difficult to find out) | System Level | Lack of care coordination | 2.5% |
| 3 | Poor communication with the primary care doctor | System Level | Lack of care coordination | 0.6% |
| 2 | From a quality reporting perspective and costs attributed to the rheumatologist, screening may add additional burden | System Level | Financial barriers | 0.6% |
| 1 | Difficult to correlate diagnosis with the screening order for lipid profile | System Level | Financial barriers | 0.0% |
| 2 | The required insurance limitations in repeating labs when trying to screen | System Level | Financial barriers | 1.2% |
| 2 | Sometimes lab cost can be an issue for patients | System Level | Financial barriers | 0.6% |
| 3 | Financial issues related to the cost of lab testing - not everyone has sufficient insurance | System Level | Financial barriers | 0.6% |
