# Supplementary material for: Views of primary care physicians and rheumatologists regarding screening and treatment of hyperlipidemia among patients with rheumatoid arthritis
Source: BMC Rheumatol. 2020 Mar 5;4:14. doi: 10.1186/s41927-020-0112-5 (PMC7057468; doi:10.1186/s41927-020-0112-5)
Supplement: Supplementary file 2 — Additional file 2: Table S2. Primary care physicians’ responses to “What are some of the things that make it difficult for you to screen for hyperlipidemia in your RA patients?” [file 41927_2020_112_MOESM2_ESM.docx]

**Table S2.** Primary care physicians’ responses to “What are some of the things that make it difficult for you to screen for hyperlipidemia in your RA patients?”

| **Group** | **Statement** | **Category** | **Sub-Category** | **% of total points** |
| --- | --- | --- | --- | --- |
| 3 | Patients are concerned about taking more medicines, and can refuse tests [polypharmacy] | Patient Level | Barriers from the patient | 0.0% |
| 2 | Many rheumatological medications cause side effects that affect our ability to treat and might deter us from screening | Patient Level | Complexity of RA and its treatment | 0.8% |
| 3 | Changing complexity of disease pattern - lipid levels change inversely with the active marker crp | Patient Level | Complexity of RA and its treatment | 2.5% |
| 1 | May not be an appropriate time to screen due to the side effects of RA medications (e.g. prednisone) | Patient Level | Complexity of RA and its treatment | 4.2% |
| 2 | Patients with RA are often on steroids which impacts their lipid profile | Patient Level | Complexity of RA and its treatment | 0.0% |
| 1 | Active inflammation at any point may increase lipids, difficult to decide the correct time to screen | Patient Level | Complexity of RA and its treatment | 1.7% |
| 1 | If patients are on biologics many other lab values must be looked at, cholesterol can be overlooked | Patient Level | Complexity of RA and its treatment | 0.0% |
| 1 | Difficulty drawing blood due to previous IVs and infusions | Patient Level | Multiple blood draws | 0.8% |
| 3 | Patients don't want to repeat labs if they have had other blood withdrawals | Patient Level | Multiple blood draws | 0.0% |
| 1 | Some patients aren't fasting at the time of visit, and are reluctant to return fasting | Patient Level | Multiple blood draws | 4.2% |
| 2 | Patients say that their rheumatologist check their blood so often that they don't want it checked in the PCP office | Patient Level | Multiple blood draws | 3.3% |
| 1 | RA patients don't know that CVD kills them, so they don't ask for the test | Patient Level | Patients' lack of awareness of CVD risk | 0.0% |
| 3 | Lack of patient awareness of importance of morbidity and mortality of atherosclerosis in the RA disease state [resistance to accepting primary care intervention] | Patient Level | Patients' lack of awareness of CVD risk | 3.3% |
| 3 | Patients may not show up for follow-up visits - they are focused on RA and not on lipid levels | Patient Level | Patient prioritization of RA symptomology over preventive measures | 2.5% |
| 2 | When patients are in a lot of pain, you may not get to address health maintenance in a visit | Patient Level | Patient prioritization of RA symptomology over preventive measures | 6.7% |
| 2 | Arthritis patients are often focused on their chronic disease and aren't interested in screening for long term risk conditions | Patient Level | Patient prioritization of RA symptomology over preventive measures | 0.0% |
| 1 | Patients with multiple medications frequently aren't compliant with all of their medications, and I am reluctant to check for lipids because of likely non-adherence | Patient Level | Patient already on multiple medications | 0.0% |
| 2 | Oftentimes patients are worried about adding more medicines and don't want to screen | Patient Level | Patient already on multiple medications | 0.0% |
| 3 | Patient prefers not to do labs if not fasting | Patient Level | Patient already on multiple medications | 0.0% |
| 1 | Patients may not come in regularly for routine testing | Patient Level | Poor patient compliance with medical care | 5.8% |
| 2 | May not be showing up to see me as frequently as their rheumatologist; less opportunity to screen | Patient Level | Poor patient compliance with medical care | 3.3% |
| 3 | Patients are resistant after learning about potential side effects [possibility of pain] | Patient Level | Side effects of statins and drug interactions with statins | 3.3% |
| 3 | Interactions of medications, e.g. impact of statins on myalgia, can adversely affect patient compliance | Patient Level | Side effects of statins and drug interactions with statins | 1.7% |
| 1 | Many patients routinely get their lipids tested by their rheumatologist, I assume cholesterol is being monitored by the specialist | Physician Level | Conflict regarding ownership of hyperlipidemia management | 8.3% |
| 2 | We ask patients to ask their rheumatologist for blood work, but there is a potential for that not to happen | Physician Level | Conflict regarding ownership of hyperlipidemia management | 2.5% |
| 1 | Lack of provider knowledge about drug interactions between statins and RA meds | Physician Level | Lack of physician knowledge about RA | 0.8% |
| 2 | I don't screen if I don't believe there is a benefit to treating it | Physician Level | Lack of physician knowledge about RA | 0.0% |
| 3 | Time constraints on practice; can be overlooked due to addressing other patient concerns | Physician Level | Lack of time | 1.7% |
| 2 | If the patient has not fasted, I won't do the test | Physician Level | Lack of training and knowledge of hyperlipidemia guidelines | 0.0% |
| 2 | Lack of clear guidelines on how to interpret and classify high lipids | Physician Level | Lack of training and knowledge of hyperlipidemia guidelines | 0.8% |
| 3 | No specific guidelines for lipid goals | Physician Level | Lack of training and knowledge of hyperlipidemia guidelines | 10.0% |
| 3 | Recommendations on screening don't specify how often interval testing should occur | Physician Level | Lack of training and knowledge of hyperlipidemia guidelines | 6.7% |
| 3 | Difficult to identify when to start screening for younger patients [Appropriate age to start screening] | Physician Level | Lack of training and knowledge of hyperlipidemia guidelines | 1.7% |
| 1 | Competing priorities such as symptom control and lack of time make it difficult to include screening in a visit | Physician Level | Physician prioritization of RA symptomology over preventive measures | 7.5% |
| 2 | Provider has a number of competing priorities that detracts from primary screening | Physician Level | Physician prioritization of RA symptomology over preventive measures | 2.5% |
| 3 | Fractured care between pcps and rheumatologists; can be difficult to determine who is responsible for screening and implementing therapy | System Level | Lack of care coordination | 7.5% |
| 3 | We don't have access to records of prior labs; difficult to tell if patient is already on treatment | System Level | Lack of care coordination | 2.5% |
| 3 | Patients may be seen by providers at other systems making it difficult to track care | System Level | Lack of care coordination | 1.7% |
| 1 | Cost of multiple labs can be high | System Level | Financial barriers (limited insurance coverage, cost of repeating labs) | 0.0% |
| 3 | Questionable as to whether or not lipid panel would be covered by RA diagnosis code | System Level | Financial barriers (limited insurance coverage, cost of repeating labs) | 0.0% |
| 3 | Patients are resistant to change regimes because of the cost of newer drug | System Level | Financial barriers (limited insurance coverage, cost of repeating labs) | 0.0% |
| 1 | When RA is compared to other chronic conditions such as diabetes, there is a lack of incentive for physicians to screen and/or control | System Level | Lack of financial incentive for screening | 1.7% |
