# Supplementary material for: Views of primary care physicians and rheumatologists regarding screening and treatment of hyperlipidemia among patients with rheumatoid arthritis
Source: BMC Rheumatol. 2020 Mar 5;4:14. doi: 10.1186/s41927-020-0112-5 (PMC7057468; doi:10.1186/s41927-020-0112-5)
Supplement: Supplementary file 3 — Additional file 3: Table S3. Rheumatologists’ responses to “What are some of the reasons why you may choose not to treat these patients for hyperlipidemia?” [file 41927_2020_112_MOESM3_ESM.docx]

**Table S3.** Rheumatologists’ responses to “What are some of the reasons why you may choose not to treat these patients for hyperlipidemia?”

| **Group** | **Statement** | **Category** | **Sub-Category** | **% of total points** |
| --- | --- | --- | --- | --- |
| 3 | Patients aren't enthusiastic about taking another medication | Patient Level | Patient already on multiple medications | 2.5% |
| 1 | Anti-lipidemic agents confound ability to identify the toxicity of RA medicines | Patient Level | Side effects and RA drug interactions | 0.6% |
| 2 | Some drugs are known to induce hyperlipidemia and a rheumatologist may take a wait and see approach to that elevated level | Patient Level | Side effects and RA drug interactions | 0.0% |
| 2 | They may have side effects to medications in the past | Patient Level | Side effects of statins | 0.0% |
| 3 | Concerns about side effects | Patient Level | Side effects of statins | 2.5% |
| 2 | Elevated liver functions may have occurred in the past | Patient Level | Side effects of statins | 0.0% |
| 3 | Another specialist may be the more appropriate physician to treat this problem | Physician Level | Conflict regarding ownership of hyperlipidemia management | 4.9% |
| 1 | Approaches to treating hyperlipidemia also involves non-pharmacologic approaches outside the domain of a rheumatologist | Physician Level | Conflict regarding ownership of hyperlipidemia management | 0.0% |
| 1 | Don't want to cross into another specialist's domain | Physician Level | Conflict regarding ownership of hyperlipidemia management | 1.9% |
| 2 | Don't want to take ownership and step on the toes of the PCP | Physician Level | Conflict regarding ownership of hyperlipidemia management | 3.1% |
| 2 | Hyperlipidemia management is not within the scope of rheumatology practice | Physician Level | Conflict regarding ownership of hyperlipidemia management | 3.7% |
| 3 | If I started, then responsibility for management would shift to me | Physician Level | Conflict regarding ownership of hyperlipidemia management | 1.9% |
| 2 | If the patient needs to be re-screened, need to refer to the PCP | Physician Level | Conflict regarding ownership of hyperlipidemia management | 1.2% |
| 2 | Need to respect the decision making of the ordering MD | Physician Level | Conflict regarding ownership of hyperlipidemia management | 1.9% |
| 1 | PCP may already have a plan in place | Physician Level | Conflict regarding ownership of hyperlipidemia management | 4.3% |
| 3 | Reluctant to start treatment without approval from the primary doctor | Physician Level | Conflict regarding ownership of hyperlipidemia management | 1.9% |
| 1 | Slippery slope into treatment responsibilities for all cardiovascular risks | Physician Level | Conflict regarding ownership of hyperlipidemia management | 6.2% |
| 1 | Treating someone with medication requires me to observe and deal with side effects or complications | Physician Level | Conflict regarding ownership of hyperlipidemia management | 4.9% |
| 1 | Usually not primary reason for referral | Physician Level | Conflict regarding ownership of hyperlipidemia management | 1.9% |
| 3 | May be better ways to decrease their risk | Physician Level | Focus only in RA | 2.5% |
| 3 | Patient will benefit more from treatment of their RA than lipids | Physician Level | Focus only in RA | 3.1% |
| 1 | Rheumatologist wants to lower the risk by treating RA | Physician Level | Focus only in RA | 0.6% |
| 1 | Not enough time to spend on anything other than RA, a complex disease | Physician Level | Lack of time | 4.3% |
| 2 | Time constraints for the office visit | Physician Level | Lack of time | 4.9% |
| 3 | The current complexity of their medical management | Physician Level | Lack of time | 1.9% |
| 3 | Don't feel as comfortable managing statins (expertise and experience) | Physician Level | Lack of training and knowledge of hyperlipidemia guidelines | 4.9% |
| 1 | Lack of current training in treating hyperlipidemia | Physician Level | Lack of training and knowledge of hyperlipidemia guidelines | 8.6% |
| 2 | Lack of updated knowledge about the treatment of hyperlipidemia | Physician Level | Lack of training and knowledge of hyperlipidemia guidelines | 12.3% |
| 1 | More cost-effective and often better care option to refer the patient to a qualified specialist | Physician Level | Lack of training and knowledge of hyperlipidemia guidelines | 1.2% |
| 1 | Rheumatologists are less familiar with the possible side effects of hyperlipidemia medicines | Physician Level | Lack of training and knowledge of hyperlipidemia guidelines | 1.2% |
| 2 | Rheumatologists don't know the nuances of lipid-lowering management | Physician Level | Lack of training and knowledge of hyperlipidemia guidelines | 3.7% |
| 3 | Concern about the cost of additional medication | System Level | Financial barriers | 0.0% |
| 1 | Patients are often resistant to adding another medication due to cost, insurance restrictions, and side effects | System Level | Financial barriers | 4.3% |
| 2 | The cost of care will increase due to CMS rules | System Level | Financial barriers | 1.2% |
| 1 | Lack of ancillary support for follow-up [future tests, etc.] | System Level | Financial barriers | 0.6% |
| 2 | Adds a burden to the staff for managing hyperlipidemia treatments | System Level | Limited clinic staff support | 1.2% |
