# Supplementary material for: Views of primary care physicians and rheumatologists regarding screening and treatment of hyperlipidemia among patients with rheumatoid arthritis
Source: BMC Rheumatol. 2020 Mar 5;4:14. doi: 10.1186/s41927-020-0112-5 (PMC7057468; doi:10.1186/s41927-020-0112-5)
Supplement: Supplementary file 4 — Additional file 4: Table S4. Primary care physicians’ responses to “What are some of the reasons why you may choose not to treat these patients for hyperlipidemia?” [file 41927_2020_112_MOESM4_ESM.docx]

**Table S4.** Primary care physicians’ responses to “What are some of the reasons why you may choose not to treat these patients for hyperlipidemia?”

| **Group** | **Statement** | **Category** | **Sub-Category** | **% of total points** |
| --- | --- | --- | --- | --- |
| 1 | Would not prioritize prevention of CVD if the patient is already very sick [limited life expectancy] | Patient Level | Comorbidities | 2.5% |
| 1 | Patient can be too sick to start a new medication (e.g. RA flare) | Patient Level | Comorbidities | 0.0% |
| 2 | Patients may be too sick to benefit from CVD reduction (difficulty evaluating life expectancy) | Patient Level | Comorbidities | 0.0% |
| 3 | Reluctant to treat patients with multiple comorbidities [diabetes, obesity, history of high cholesterol, hormonal changes as patient ages] | Patient Level | Comorbidities | 2.5% |
| 3 | Would be reluctant to start a statin in a patient that has chronic hepatitis or moderate alcohol use | Patient Level | Comorbidities | 1.7% |
| 3 | Concurrent elevation in liver associated enzymes or liver function tests due to RA meds or RA disease state | Patient Level | Complexity of RA and its treatment | 5.8% |
| 1 | Patient may be reluctant to take a new medication due to number of meds they are already on | Patient Level | Patient already on multiple medications | 4.2% |
| 2 | RA patients are reluctant to take more medications | Patient Level | Patient already on multiple medications | 4.2% |
| 2 | Patient refusal to take a statin | Patient Level | Patients' lack of awareness of CVD | 1.7% |
| 3 | Patient may have little concern for cardiovascular risk factors compared to devastating RA condition | Patient Level | Priority of RA symptomology over preventive measures | 2.5% |
| 3 | Patient may refuse treatment despite educating the patient about the importance of treatment for hyperlipidemia | Patient Level | Priority of RA symptomology over preventive measures | 1.7% |
| 1 | If the patient experiences a lot of myalgia or myopathy, a statin may exacerbate those symptoms | Patient Level | Side effects of statins and drug interactions with statins | 9.2% |
| 1 | If a patient already has elevated liver function test from disease or other medications, I would be reluctant to prescribe a statin | Patient Level | Side effects of statins and drug interactions with statins | 5.0% |
| 1 | Interactions with other medications a patient is on | Patient Level | Side effects of statins and drug interactions with statins | 3.3% |
| 1 | Patient prior intolerance to medication | Patient Level | Side effects of statins and drug interactions with statins | 0.0% |
| 1 | Statins are contraindicated during pregnancy | Patient Level | Side effects of statins and drug interactions with statins | 0.0% |
| 2 | Lipid therapy might be contraindicated due to side effects of other medications | Patient Level | Side effects of statins and drug interactions with statins | 4.2% |
| 2 | Reluctant to use statins on patients who can not tolerate statin myopathy | Patient Level | Side effects of statins and drug interactions with statins | 0.0% |
| 3 | Allergy to statin or high baseline CK would be a reason to avoid therapy | Patient Level | Side effects of statins and drug interactions with statins | 4.2% |
| 3 | Would avoid treatment during pregnancy | Patient Level | Side effects of statins and drug interactions with statins | 0.0% |
| 1 | Concern about the long-term impact of statin on cognitive abilities | Patient Level | Side effects of statins and drug interactions with statins | 0.0% |
| 3 | Fear of polypharmacy; trying to avoid detrimental drug-drug or drug-disease interactions | Patient Level | Side effects of statins and drug interactions with statins | 6.7% |
| 2 | Uncomfortable about drug-drug interactions | Patient Level | Side effects of statins and drug interactions with statins | 6.7% |
| 3 | May not treat due to concerns about the risk and/or side effects of available treatments | Patient Level | Side effects of statins and drug interactions with statins | 3.3% |
| 1 | Clinical calculators for younger RA patients can underestimate CVD risk | Physician Level | Lack of training and knowledge of hyperlipidemia guidelines | 5.0% |
| 2 | Lack of clear guidelines on LDL goals and duration of treatment | Physician Level | Lack of training and knowledge of hyperlipidemia guidelines | 0.0% |
| 3 | There is no specific guideline of when to start a patient on lipids [age, lipid level, etc.] | Physician Level | Lack of training and knowledge of hyperlipidemia guidelines | 10.0% |
| 3 | Many people use ASCVD calculator which would likely underestimate patient cardiovascular risk | Physician Level | Lack of training and knowledge of hyperlipidemia guidelines | 3.3% |
| 1 | If the patient is already on prednisone or at risk for diabetes, concern that a statin will increase that risk | Physician Level | Lack of training and knowledge of hyperlipidemia guidelines | 0.0% |
| 1 | Would not prescribe to a non-fasting patient | Physician Level | Lack of training and knowledge of hyperlipidemia guidelines | 0.0% |
| 3 | Would prefer if the rheum or cardiologist would treat the patient for high lipid levels, especially if they are on new medications for RA | Physician Level | Conflict regarding ownership of HL management | 0.0% |
| 2 | Reluctance to push behavioral interventions such as diet or exercise for patients experiencing chronic pain | Physician Level | Difficulty implementing lifestyle modifications for patients with pain | 1.7% |
| 2 | Lack of time to make a shared decision | Physician Level | Lack of time | 0.0% |
| 2 | Competing priorities at the visit | Physician Level | Lack of time | 0.0% |
| 3 | Time constraints - too many other issues to discuss to fully explain rationale for treatment and potential side effects | Physician Level | Lack of time | 0.8% |
| 1 | Recommending exercise may be more beneficial to controlling both lipids and RA | Physician Level | Prioritize non-pharmacologic measures | 1.7% |
| 3 | Prefer to try diet and exercise program first | Physician Level | Prioritize non-pharmacologic measures | 2.5% |
| 1 | Cost of the medication can be prohibitive for some patients | System Level | Financial barriers | 0.0% |
| 1 | Poor communication between EHRs and concern that patient would be lost to follow up makes me reluctant to prescribe a statin | System Level | Lack of care coordination | 2.5% |
| 1 | Miscommunication between practices, sometimes assume that a rheum will prescribe after testing for lipids | System Level | Lack of care coordination | 1.7% |
| 2 | Inability to reach the rheumatologist to discuss | System Level | Lack of care coordination | 1.7% |
| 3 | Difficulty in communications between rheum and pcp - getting records on blood levels, potential interactions, etc. | System Level | Lack of care coordination | 0.0% |
